# Large Solid Right Atrial Thrombus Treated by AngioVac Catheter-Based Suction Thrombectomy

**DOI:** 10.1155/2018/7904064

**Published:** 2018-11-08

**Authors:** Jacob Schultz, Asger Andersen, Erik Lerkevang Grove, Lars Bo Ilkjær, Jens Erik Nielsen-Kudsk

**Affiliations:** ^1^Department of Cardiology, Aarhus University Hospital, Denmark; ^2^Department of Clinical Medicine, Faculty of Health, Aarhus University, Denmark; ^3^Department of Cardiothoracic and Vascular Surgery, Aarhus University Hospital, Denmark

## Abstract

We present the successful treatment of a large solid right atrial thrombus by the catheter-based suction embolectomy system AngioVac® (AngioDynamics, NY, USA). A previously healthy 60-year-old male was referred with acute pulmonary embolism, a large deep vein thrombus and a large right atrial thrombus. After one week of anticoagulation, the size of the atrial thrombus was unaltered, and the patient was treated by catheter-directed embolectomy using the AngioVac system. The solid thrombus occluded the catheter during the procedure. With the vacuum maintained, the occluded catheter was removed from the patient and the thrombus mass was removed. The remaining atrial thrombus was successfully removed by suction after the reinsertion of the catheter. The patient recovered well and was discharged 7 days after the procedure. The therapy was safe and minimally invasive.

## 1. Introduction

Catheter-based aspiration therapy has shown promise in the treatment of venous thromboembolism (VTE). The AngioVac system consists of a large (22F) bore catheter with an expandable funnel-shaped distal tip. The catheter is connected to an extracorporeal venous-venous bypass system, allowing for filtration of aspirated blood from thrombi before reintroducing the blood into the systemic circulation [1]. The system is primarily used in the peripheral circulation, but it is increasingly utilized in the treatment of central venous thrombi and other masses [2–5]. The majority of published cases report successful removal of larger thrombi [3, 4]. Cases with firmer and/or adherent thrombi have meanwhile posed a challenge [4]. Here, we present the successful treatment of a large solid right atrial thrombus with the catheter-based suction embolectomy system AngioVac.

## 2. Case Presentation

A previously healthy 60-year-old male was referred to the outpatient clinic due to atrial fibrillation. The patient reported pain in the lower left leg for 3 weeks followed by right-sided chest pain and dyspnea for 2 weeks. Transthoracic echocardiography (TTE) revealed a dilated right atrium (RA) with a large longitudinal thrombus (1–1.5 cm × 15–20 cm) fluctuating through the tricuspid valve (Video 1). The patient was stable and had no signs of right or left ventricular strain. Treatment with rivaroxaban 15 mg × 2 was initiated, and he was admitted to our center with suspected multilevel VTE: deep venous thrombosis (DVT), RA thrombus, and acute pulmonary embolism (PE). Computed tomography confirmed PE in the lower right pulmonary artery with associated pleural effusion. TTE and transesophageal echocardiography (TEE) confirmed the RA thrombus. Ultrasound revealed a large DVT in the left femoral vein stretching from the popliteal to the iliac vein. The patient was switched from rivaroxaban to unfractionated heparin (UFH) 5000 IE bolus followed by infusion starting at 1000 IE/hour and monitored by APTT. APPT remained in the lower range (maximum 77) treatment despite increasing doses of UFH to a maximum dose of 1900 IE/hour. After 3 days of UFH treatment, there was no regression of RA thrombus on TTE. The thrombus appeared to be attached in a thin fibrotic pedicle to the area between the superior vena cava and RA (Video 2). No persistent foramen ovale or atrial septal defect was found. Due to the large size and thin attachment, the risk of a possibly fatal PE was considered significant. As there were no regression in thrombus despite 7 days of anticoagulation treatment, it was decided to refer the patient for catheter-based embolectomy using the AngioVac system.

Preprocedural planning included a new ultrasound of the lower extremities that confirmed regression of thrombus in the lower veins bilaterally. This allowed for a femoral venous-venous access. The procedure was performed in a hybrid suite with a multidisciplinary team from interventional cardiology and thoracic surgery enabling fast conversion to extracorporeal circulation and surgical embolectomy if needed. The patient was placed in general anesthesia, and the procedure was guided by fluoroscopy and continuous TEE. A 26F dry-seal sheath (Gore Medical®) was placed in the right femoral vein to accommodate the AngioVac cannula and an 18F reinfusion cannula in the left femoral vein. A venous sheath was placed in the left external jugular vein to allow for conversion to a jugular approach, insertion of an adjunctive catheter, or a temporary v. cava filter if needed. Furthermore, a 6F sheath was placed in the right femoral artery allowing easy conversion to venous-arterial bypass.

**Figure 1:**
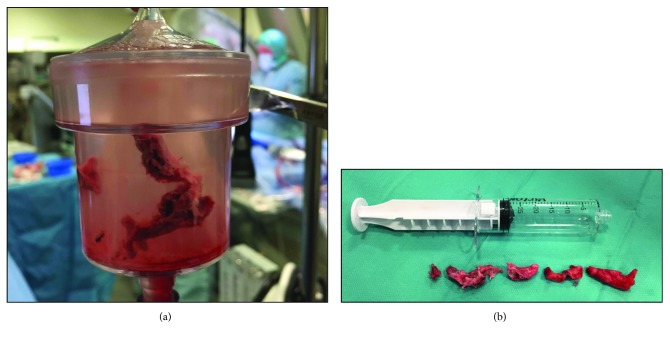
(a) Photo of removed thrombus trapped in the filter. (b) Photo of all removed thrombus fragments.

The catheter was placed in the inferior vena cava. The funnel-shaped tip was then opened, and the centrifugal pump started. Using a flow of 3.5 L/min, the catheter was slowly moved towards the RA, and part of the thrombus was sucked into the tip. The solid thrombus occluded the catheter and stopped the flow completely. With the vacuum maintained, the occluded catheter was removed from the patient, and the thrombus was removed from the catheter (Video 3). The catheter was reintroduced to the RA, and this time thrombus material was sucked out and into the filter (Figure 1(a)). A small thrombus of 5 × 8 mm remained attached despite significant suction (Video 4). Sheaths were removed and venous access closed by percutaneous suture and the arterial access by Angio-Seal®. The thrombus fragments removed by the procedure measured a total of 15 × 1 cm and consisted of heterogeneous solid older thrombus material **(** Figure 1(b)), which was confirmed by the pathologist after the procedure.

The patient recovered well after the procedure. He was treated with low molecular weight heparin (7500 IEx2) and was discharged 7 days postprocedure to follow-up in the outpatient PE clinic.

## 3. Discussion

The AngioVac system has previously been used for central venous thrombi. Despite being the largest catheter available, large thrombi may however still occlude the lumen and stop the circuit. While the substantial vacuum can deform or fragment fresh thrombi, this may not be the case with more chronic thrombi. In the present case, the thrombus proved more solid than expected, and the catheter was obstructed. We successfully removed the catheter and thrombus *en bloc*. Despite the vacuum, thrombus material may dislodge and embolize to the pulmonary circulation. While the potential of the technique is promising, we advocate that procedures are only performed in tertiary centers with access to acute surgical embolectomy, preferably using a multidisciplinary approach.

We describe the successful treatment of a large solid right atrial thrombus with the catheter-based suction embolectomy system AngioVac. The therapy was safe and minimally invasive.

## Supplementary Materials

Supplementary 1 Video 1: transthoracic echocardiography showing a dilated right atrium with a large longitudinal thrombus fluctuating through the tricuspid valve.

Supplementary 2 Video 2: transoesophageal echocardiography of thrombus adherent in right atrium prior to intervention.

Supplementary 3 Video 3: video of obstructing thrombus material removed from the AngioVac catheter.

Supplementary 4 Video 4: transoesophageal echocardiography of residual thrombus adherent despite significant suction.
